# Heat Transfer Capabilities of Surface Cooling Systems for Inducing Therapeutic Hypothermia

**DOI:** 10.1089/ther.2023.0003

**Published:** 2023-09-01

**Authors:** Curtis Leclerc, Morteza Talebian nia, Gordon G. Giesbrecht

**Affiliations:** ^1^ Faculty of Kinesiology and Recreation Management, University of Manitoba, Winnipeg, Canada.; ^2^ Department of Anesthesia and Emergency Medicine, Faculty of Medicine, University of Manitoba, Winnipeg, Canada.

**Keywords:** cerebral ischemia, hypothermia, skin cooling

## Abstract

Therapeutic hypothermia (TH) is used to treat patients with cerebral ischemia. Body surface cooling provides a simple noninvasive method to induce TH. We compared three surface cooling systems (Arctic Sun with adhesive ArcticGel pads [AS]); Blanketrol III with two nonadhesive Maxi-Therm Lite blankets [BL]); and Blanketrol III with nonadhesive Kool Kit [KK]). We hypothesized that KK would remove more heat due to its tighter fit and increased surface area. Eight subjects (four females) were cooled with each system set to 4°C outflow temperature for 120 minutes. Heat loss, skin and esophageal temperature, and metabolic heat production were measured. Skin temperature was higher with KK (*p* = 0.002), heat loss was lower with KK in the first hour (*p* = 0.014) but not after 120 minutes. Heat production increased similarly with all systems. Core temperature decrease was greater for AS (0.57°C) than BL (0.14°C; *p* = 0.035), but not KK (0.24°C; *p* = 0.1). Each system had its own benefits and limitations. Heat transfer capability is dependent on the cooling pump unit and the design of the liquid-perfused covers. Both Arctic Sun and Blanketrol III cooling/pump units had 4°C output temperatures. However, the Blanketrol III unit had a greater flow rate and therefore more cooling power. The nonadhesive BL and KK covers were easier to apply and remove compared with the adhesive AS pads. AS had an early transient advantage in heat removal, but this effect decreased over the course of cooling, thus minimizing or eliminating any advantage during longer periods of cooling that occur during clinical TH.

Clinical Trial Registration number: NCT04332224.

## Introduction

Cerebral ischemia occurs when myocardial infarction or stroke decreases blood flow to the brain. The resultant decrease in oxygen delivery shifts metabolism to less efficient anaerobic energy production (Martin et al., 1994) initiating a cascade of events resulting in excitotoxicity, inflammation, and free radical production (Yenari and Han, 2012; Yenari et al., 2008).

Therapy for ischemic strokes can be divided into reperfusion and neuroprotection. Reperfusion therapies are treatments that restore blood flow and effective methods include intravenous administration of thrombolytic drugs and surgical endovascular thrombectomies (El Amki and Wegener, 2017). Neuroprotective therapies are those that interrupt the cellular, biochemical, and metabolic processes that lead to brain injury during ischemia and include the use of medications, surgery, or hypothermia (Auriel and Bornstein, 2010). Therapeutic hypothermia (TH) has emerged as a strong neuroprotective therapy, and it has been validated in laboratory and clinical settings (Bernard et al., 2002; Holzer et al., 2002; Yenari and Han. 2012).

The American Heart Association, European Resuscitation Council, and the International Liaison Committee on Resuscitation all provide postresuscitation guidelines and recommendations that support the use of TH in patients postcardiac arrest (Callaway et al., 2015a; Callaway et al., 2015b; Deakin et al., 2010). These guidelines define TH as a controlled reduction of the body's core temperature to roughly 32–36°C for a minimum duration of 24 hours through a process of targeted temperature management (TTM).

The benefits of TTM have not been consistently demonstrated. An early study on patients with cardiac arrest due to ventricular fibrillation reported that after 6 months the TH group had a decreased mortality and favorable neurologic outcome (Hypothermia After Cardiac Arrest Study Group, 2022). However, a recent study (TTM2) (Dankiewicz et al., 2021) revealed that outcomes for out-of-hospital cardiac arrest were similar whether treated by normothermia or hypothermia. However, the application of a cooling cover was still recommended in case active cooling was required to combat fever. Two other studies also demonstrated no effect of TH on overall mortality rates, however, there was a higher percentage of survival with good neurologic outcome after 90 days in patients with postcardiac arrest and a nonshockable rhythm (Lascarrou et al., 2019), and better neurologic outcomes when TH was applied to patients with moderate encephalopathy (Nutma et al., 2022).

The neuroprotective properties of TH offer protection against ischemia by reducing oxygen consumption and preserving energy stores for the neurons (Erecinska et al., 2003). Preserving the brain's energy could reduce the downstream effects of ionic imbalances and reduce the risk of acidosis. TH has also been shown to mitigate damage to endothelial cells and increases in blood–brain barrier permeability (Dietrich et al., 1990); reduce hydroxyl radical production (Globus et al., 1995); and improve postischemia glucose utilization (Ginsberg et al., 1993). Furthermore, TH has shown the ability to suppress glutamate release/sensitivity in ischemic rat brains (Busto et al., 1989; Friedman et al., 2001). This could attenuate the severity of excitotoxicity during the ischemic cascade by reducing the action of degradative enzymes on neurons. It is the combination of these neuroprotective mechanisms that make TH an attractive treatment for ischemic patients.

Despite these known neuroprotective effects, forced hypothermia does have potential negative consequences including vigorous shivering, impaired drug metabolism, cold diuresis, abnormalities in electrolytes and coagulation time, and increased infection rates (Kochanek and Jackson, 2017). As well, the TTM2 study reported that TH resulted in a higher rate of arrhythmias causing hemodynamic compromise (Dankiewicz et al., 2021). In addition to the possible negative effects of cooling, TH may be difficult to achieve in adult humans because of a large mass to cool; layers of adipose, muscle, and connective tissue insulate the body from heat loss; and thermoprotective shivering heat production.

Body cooling methods are classified into either central cooling or surface cooling. Central cooling introduces cold fluids into a patient's circulation and uses conductive heat transfer to cool the core. These techniques are most effective, but they are invasive and introduce more risk for the patient (De Fazio et al., 2019). Surface cooling techniques use external noninvasive methods to induce core cooling. The effectiveness of surface cooling methods depends on the surface area and thermal conductivity of the cooling surfaces.

Two commercial surface cooling systems that are commonly used in a clinical setting are the following: (1) the Blanketrol III Hyper-Hypothermia Temperature Management System with Maxi-Therm Lite blankets (Gentherm Medical, Cincinnati) and (2) the Arctic Sun Temperature Management System with adhesive ArcticGel pads (Becton, Dickinson and Company, Mississauga). Both systems have been demonstrated to be effective in laboratory settings (Heard et al., 2010; Islam et al., 2015; Shinada et al., 2014). A new cooling cover system, the Kool Kit (KK), has been developed for the Blanketrol III cooling pump unit. The KK has a tight-fitting hood and vest to provide targeted cooling of the head and torso, as well as a single blanket to cover the anterior legs. We are unaware of any studies comparing these established systems as well as the newer KK system.

This study tested the hypothesis that the Blanketrol III with the KK would provide the most heat transfer capabilities, due to its tighter fit and increased surface area in highly perfused areas, compared with the standard Blanketrol III and Arctic Sun systems. We initially planned to compare the cooling units with subjects whose shivering heat production was pharmacologically attenuated with IV meperidine (Goheen et al., 1997; Hultzer et al., 2005; Kulkarni et al., 2019). However, due to the strain imposed by the COVID-19 pandemic on the medical community, our collaborating physicians were not available to administer the meperidine. We assumed that the unit with the most heat transfer capabilities in shivering subjects will also be most effective in nonshivering patients.

## Methods

### Subjects

The experimental protocol was approved by the University of Manitoba Education/Nursing Research Ethics Board (Protocol # HS23118 [B2019:076]) and was registered in ClinicalTrials.gov. Before participation, a signed informed consent was obtained. A sample size of 8 was calculated to achieve 90% power (*a* = 0.05, 1-tailed test; ß = 0.10; power index of 2.92) to detect a statistically significant difference (mean ± standard deviation [SD]) in total heat loss of 30 ± 29 W between cooling conditions. Thus, 8 healthy subjects (4 males and 4 females; aged 21–26 years) were enrolled in the study. There were no dropouts, and so, no further subjects were recruited.

### Measurements

Age, body fat percentage, weight, height, and body surface area were determined. Body fat percentage was measured using a body composition analyzer (InBody 270, CA). For each trial, subjects wore a swimsuit and were instrumented in an ambient laboratory temperature of ∼22°C. Esophageal temperature (T_es_) was measured with a disposable esophageal thermocouple (Mon-a-therm) inserted through the nose, to midway down the esophagus at the level of the heart. Oxygen consumption was continuously measured with a metabolic cart (Parvo Medics, Utah). Subjects wore a face mask, which collected their expired breath during the baseline and cooling period. Time at which each subject started shivering was also noted.

Cutaneous heat flux (W/m^2^) and skin temperature (°C) were measured using 12 heat flux discs (Concept Engineering). The discs (2 cm in diameter) were taped to the skin at the following sites: forehead, dorsum of the head, left anterior chest, right anterior abdomen, right scapula, left lower back, left anterior upper arm, right posterior upper arm, left anterior thigh, right posterior thigh, right anterior lower leg, and left posterior lower leg (Fig. 2). Heat flux was defined as positive when heat traversed from the skin toward the environment (i.e., heat loss). Outflow liquid temperature and flow rate of each cooling pump unit were recorded every 5 minutes from the start of cooling.

Subjective cold discomfort was determined with the Cold Discomfort Scale (CDS) on a 0- to 10-point scale in 0.5-point increments, where responses ranged from 0 (slightly cold) to 10 (unbearably cold) (Lundgren et al., 2014). Values were determined at the start of cooling and every 15 minutes until completion.

### Cooling conditions

**FIG. 1.**
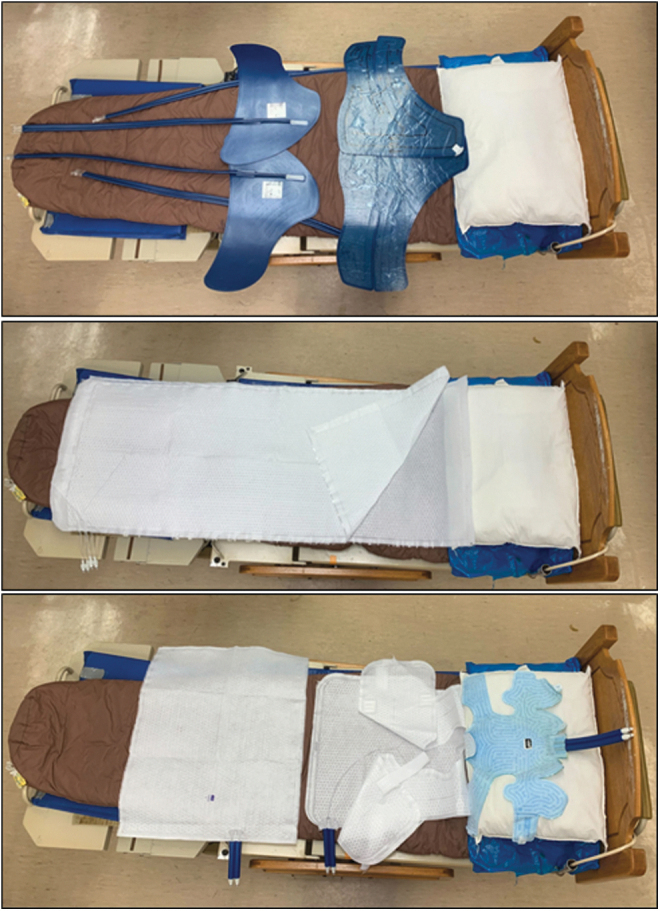
*Top*, Arctic Sun™ 5000 Temperature Management System with ArcticGel™ pads (two on the torso and two on the thighs). *Middle*, Blanketrol III Hyper-Hypothermia Temperature Management System with two Maxi-Therm Lite blankets (placed underneath and on *top* of subject). *Bottom*, Kool-Kit^®^ (including head cover, vest, and lower body blanket).

Three commercial cooling systems included the following: (1) Arctic Sun 5000 with the adhesive ArcticGel™ pads; (2) Blanketrol III with two Maxi-Therm Lite blankets; and (3) Blanketrol III with Kool Kit (Fig. 1). For each condition, the subject lay supine on a bed while the cooling covers were applied and was then covered with a cotton blanket. Cooling covers were applied according to the manufacturer's recommendations and the cooling pumps were set to their minimum liquid temperatures of 4°C. The cooling pump liquid was allowed to equilibrate with laboratory temperature and the pump/coolers were not turned on until the start of the cooling period.

### Arctic Sun with hydrogel-coated pads (AS)

**FIG. 2.**
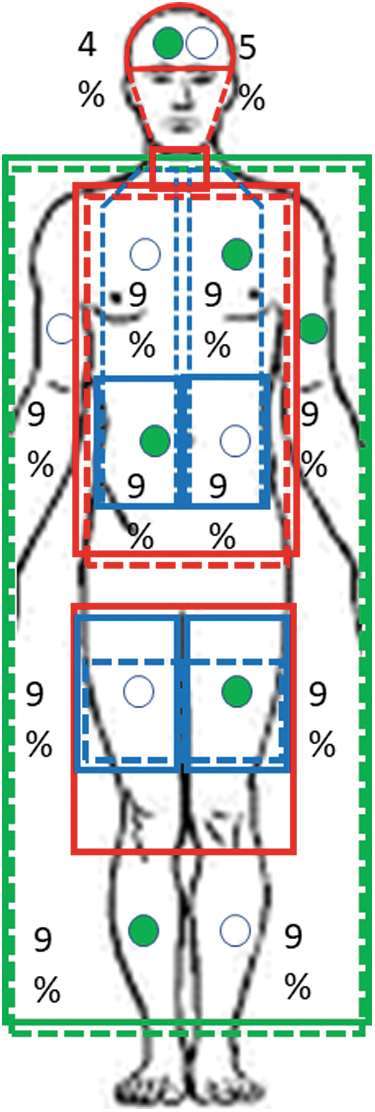
Locations of liquid-perfused covers and heat flux discs with their regional % body surface area. Covers: *solid lines* anterior, *dotted lines* posterior; *green*, Maxi-Therm Lite blankets; *red*, Kool Kit; *blue*, ArcticGel pads. Disks: *closed circles* anterior; *open circles*, posterior.

The Arctic Sun™ 5000 Temperature Management System circulates cold distilled water through four adhesive hydrogel-coated pads (ArcticGel) (Fig. 1, top; and Fig. 2, blue outlines) (Medivance, 2023). The pads are designed to cover the back and sides of the torso, abdomen, and thighs.

### Blanketrol III with Maxi-Therm Lite blankets (BL)

The Blanketrol III with Maxi-Therm Lite blankets circulates cold liquid distilled water through two whole-body nonadhesive perfused blankets (Fig. 1, middle; and Fig. 2, green outlines) (Gentherm-Medical, 2018). Each blanket extends approximately from the neck to the feet (depending on body size) and the subject lies between the blankets.

### Blanketrol III with Kook Kit (KK)

The KK is a new nonadhesive cover system developed for the Blanketrol III cooling pump. This system includes a head wrap, a vest, and a lower body blanket that extends from the waist to approximately the knees (Fig. 1, bottom; and Fig. 2, red outline).

### Protocol

Subjects were cooled three times with a different cooling system used each time. Trials were repeated with a minimum of 2 days between to minimize effects of multiple cooling trials. The order of conditions was randomly assigned to achieve a balanced design to control for any possible effects of serial cooling trials. Subjects were studied at the same time of the day to control for circadian effects. After instrumentation, the subjects lay still, and baseline measurements were recorded for 10 minutes at an ambient room temperature. Subjects were then cooled until (1) core temperature reached 35°C; (2) 120 minutes elapsed; (3) the subject wished to stop for any reason; or (4) a researcher advised termination for any reason. Subjects were then rewarmed by entering a warm bath of ∼41°C until core temperature returned to normal (36.5–37°C).

The BL and KK blankets and vest are designed so cooling liquid perfused along one side and returns on the other, thus side-to-side temperature is not uniform (e.g., the delivery side is cooler than the return side). To prevent an overall over/underestimation of measurements from either side, the delivery and return hoses to the blankets/vests were switched after ∼1 hour of cooling. This was not necessary with the AS pads.

### Data analysis

Change in core temperature (ΔT_co_) over the 120 minutes was calculated. Metabolic rate was calculated from oxygen consumption (VO_2_) by using the following equation:


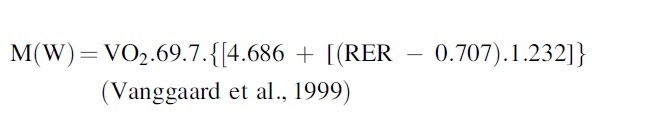


Heat flux for each site (W/site) was calculated from flux values for each transducer (W/m^2^) as follows: HF_site_ (W) = HF_disc_ (W/m^2^) × body surface area (m^2^) × regional% × 0.01. The following regional percentages were assigned based on previous work (Fig. 2) (Layton et al., 1983): forehead, 4%; dorsum of the head, 5%; left anterior chest, 9%; right anterior abdomen, 9%; right scapula, 9%; left lower back, 9%; left anterior upper arm and hand, 9%; right posterior upper arm and hand, 9%; left anterior thigh, 9%; right posterior thigh, 9%; right anterior lower leg and foot, 9%; and left posterior lower leg and foot, 9%; the missing 1% refers to the groin area. Total heat flux was calculated as well as regional heat flux from the head, upper body, and lower body according to Figure 2. Mean skin temperature was calculated as a weighted mean.

A repeated-measures two-way analysis of variance compared values from baseline and 8, 30, 60, 85, and 120 minutes of cooling). Two subjects withdrew from their trials early (at ∼85 minutes), and therefore, data from 85 minutes were analyzed to compare all subjects before their withdrawal. Data from 8 minutes were also included in the analysis due to a consistent peak at this time for one device. *Post hoc* analysis for significant differences was accomplished using Tukey's test. For cold rankings, a one-way analysis of variance on ranks (Kruskal–Wallis test) was used. Results are reported as mean ± SD; *p* ≤ 0.05 identifies statistically significant differences.

## Results

Eight healthy subjects (4 females, 4 males) participated (Table 1). They were 24 ± 2 years old; 171 ± 13 cm tall; weighed 77 ± 25 kg; had a body surface area of 1.9 ± 0.4 m^2^; and had 22% ± 9% body fat. Average cooling time for all trials was 112 ± 14 minutes (range 86–120 minutes).

**Table 1.** Subject Information

Subject	1	2	3	4	5	6	7	8	Mean	SD
Sex	F	M	M	M	M	F	F	F		
Age (years)	23	24	26	21	24	22	24	26	23.8	1.8
Height (cm)	154.5	173	184	193	170	167	160	163	170.6	12.7
Weight (kg)	58.7	79.1	104.1	123.1	64.4	71.9	50.6	66.5	77.3	24.5
BF (%)	33.9	13.4	13	26.6	13.2	34.5	15.9	24.4	21.9	9.2
BSA (m^2^)	1.52	1.91	2.26	2.52	1.8	1.79	1.48	1.7	1.9	0.4

SD, standard deviation.

### Heat flux

**FIG. 3.**
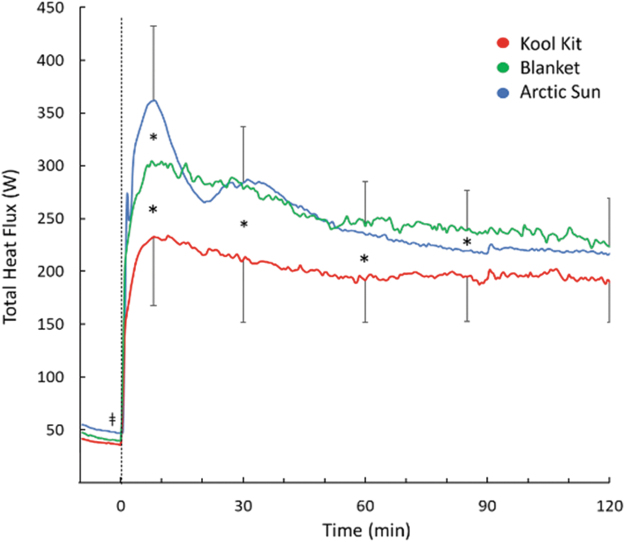
Total heat flux during 120 minutes of cooling with three cooling systems. Time 0 indicates start of cooling. For clarity, SD bars are only included for *top* and *bottom lines*. *Separates values that are significantly different from each other (*p* < 0.05). SD, standard deviation.

Total heat loss was transiently highest with AS at the start of cooling (*p* = 0.04; Fig. 3). Heat loss for BL and AS was greater than for KK throughout the first 60 minutes (*p* = 0.003) and there were no differences between systems by the end of cooling (*p* = 0.1).

**FIG. 4.**
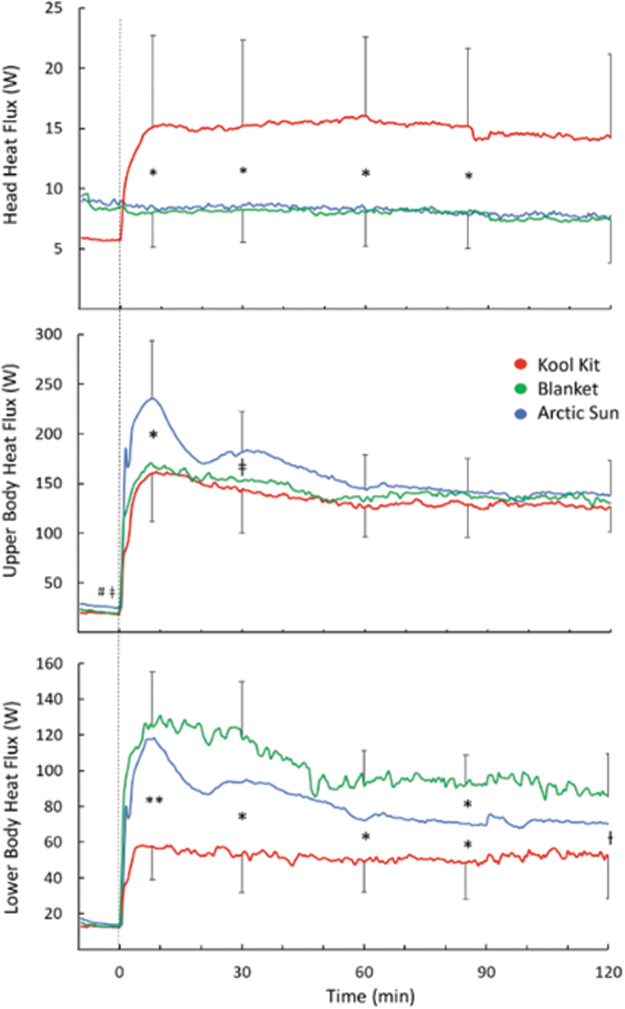
Regional heat flux for the head, upper body, and lower body during 120 minutes of cooling for three conditions. Time 0 indicates the start of cooling. For clarity, SD bars are only included for *top* and *bottom lines*. *Separates values that are significantly different (*p < 0.05; **p < 0.001); ƚ BL > KK (*p* < 0.03); ǂAS > KK (*p* < 0.05). AS, Arctic Sun; BL, blankets; KK, Kool Kit.

Head heat loss for KK was higher for the first 90 minutes of the cooling period (*p* = 0.016; Fig. 4 top); this difference was not significant at the end of cooling (*p* = 0.051). Upper body heat loss was transiently greater with AS (*p* = 0.004), but there were no differences during the last 60 minutes of cooling (*p* = 0.31; Fig. 4 middle). Lower body heat loss was greater with BL than KK throughout the cooling period (*p* < 0.001 at 10 minutes; *p* = 0.015 for the remaining time) and greater with AS than KK throughout cooling (*p* = 0.018) except at 120 minutes (*p* = 0.23) (Fig. 4 bottom).

### Temperature and heat production

**FIG. 5.**
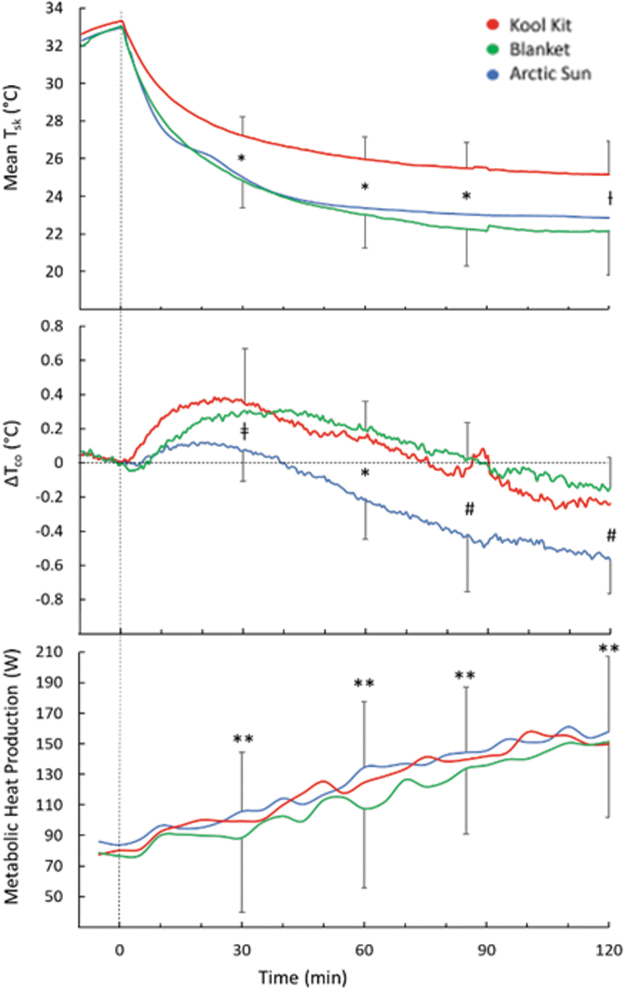
Mean skin temperature (T_sk_) (*top*); change in core temperature (ΔT_co_) (*middle*), and metabolic heat production (*bottom*) during 120 minutes of cooling with three cooling systems. Time 0 indicates the start of cooling. For clarity, SD bars are only included for *top* and *bottom lines*. *Separates values that are significantly different; ƚKK > BL; ǂKK > AS; #AS < BL; **different than baseline, (*p* < 0.05).

Mean T_sk_ was about 3°C lower with BL and AS than KK from 30 to 120 minutes of cooling (*p* = 0.003) (Fig. 5 top). Changes in T_co_ during cooling are presented in Figure 5 middle. T_co_ initially rose in all conditions. At the end of cooling, the decrease in T_co_ for AS (0.57°C) was greater than for BL (0.14°C; *p* = 0.035) but not KK (0.24°C; *p* = 0.1). There were no interactions between body weight and sex on the comparative changes in T_co_. In all conditions, metabolic heat production rose steadily and significantly from baseline (*p* < 0.05), but there were no intercondition differences (*p* = 0.96; Fig. 5 bottom).

**FIG. 6.**
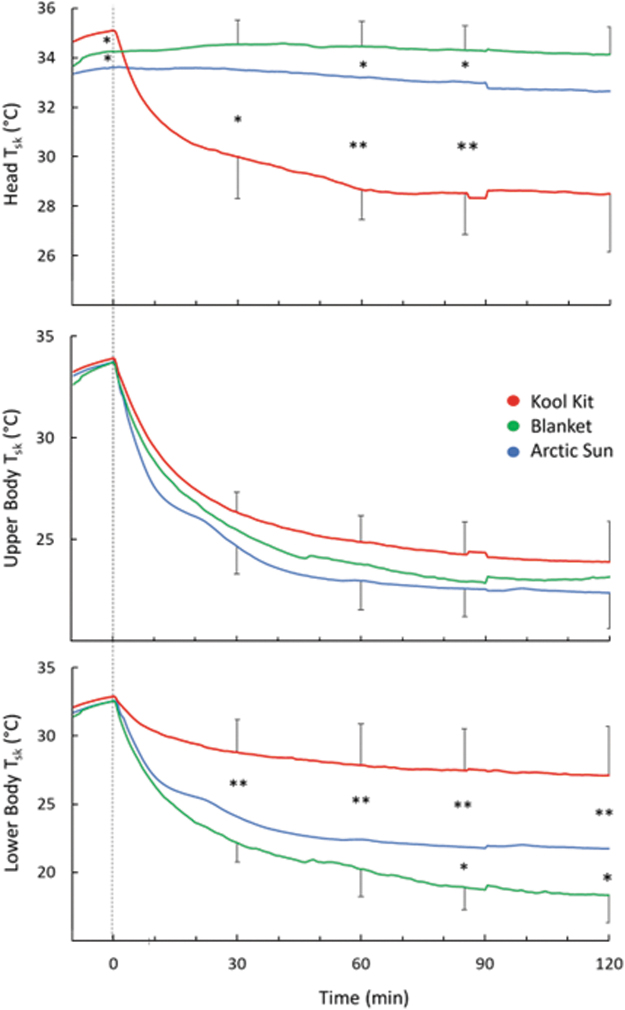
Comparison of head, upper body, and lower body T_sk_. Time 0 indicates the start of cooling. For clarity, SD bars are only included for *top* and *bottom lines*. *Separates values that are significantly different (*p < 0.05; **p < 0.01).

Head skin temperature was lower with KK than both AS and BL throughout cooling (*p* = 0.002) (Fig. 6, top). Upper body skin temperature decreased similarly with the three conditions throughout cooling (*p* = 0.35; Fig. 6, middle). Lower body skin temperature was lower with BL and AS than KK throughout cooling (*p* = 0.01; Fig. 6, bottom).

### Outflow liquid temperature and flow rate

Outflow liquid temperature was similar in all conditions, decreasing rapidly for 30 minutes and then more gradually for the remaining 90 minutes of cooling, from about 23°C to 5°C. The cooling pump flow rate was only displayed by the Arctic Sun unit. Mean flow quickly and transiently increased to 2.5 L/min and then decreased to a steady rate of about 1.7 L/min; this explains the early transient increase in heat loss with this system. The Blanketrol III cooling pump unit used a visual indicator wheel to confirm there was liquid flow but did not provide a quantitative flow rate.

### Subjective measures of cold

Cold discomfort during cooling increased similarly in all three conditions with a final combined mean value of 8.3 where 10 is “unbearably cold” (*p* = 0.7). There were no significant differences in cold ranking between the three cooling conditions (*p* = 0.84).

## Discussion

While several previous investigations have separately demonstrated the efficacy of the AS (Badjatia et al., 2022; Badjatia et al., 2009; Heard et al., 2010; Perez et al., 2020; Pittl et al., 2013; Tømte et al., 2011) and BL (Kaikaew et al., 2018; Laptook et al., 2014), this is the first study that we are aware of that compared the performance of both of these established cooling systems as well as the newer KK system. Although the AS system had a brief early advantage in heat removal, it performed similarly to BL in removing more heat than KK throughout the remaining cooling period. At the end of cooling, the decrease in core temperature with AS was 0.3–0.4°C more than with KK and BL.

Heat transfer capability depends on the cooling capacity of the cooling pump unit and the surface area and characteristics of the covers. The cooling capacity of a cooling pump unit depends on liquid temperature and liquid flow rate. Both the Arctic Sun and Blanketrol III units cooled the liquid to 4°C. Flow rates cannot be compared directly since only the AS unit displayed flow rate. However, manufacturer specifications indicate that flow rates should be >2.3 L/min for the Blanketrol III and >1.7 L/min for the Arctic Sun. The AS system performed within specifications (e.g., ∼1.7 L/min) and it is assumed that the Blanketrol unit also performed within specifications. Therefore, the Blanketrol cooling unit likely has greater cooling power.

The heat transfer capability of the covers is positively correlated to surface area, thermal conductivity (k) of the surface material, tightness of fit, and adhesion of the cover to the skin. The surface material properties impact the heat transfer capabilities of the covers by affecting thermal conductivity (k). The k value for the hydrogel used in the ArcticGel pads was ∼0.33–0.51 W/m°K, while the k value for the plastic and nonwoven fabric of BL and KK covers was ∼0.14–0.36 W/m°K (Hansen and Bernier, 1972; Tang et al., 2017). Thus, thermal conductivity of the AS covers is two to three times that of the BL and KK covers.

The tightness of fit impacts the heat transfer capabilities of the covers by affecting the average distance between the skin and the cover. Covers with a tighter fit to the skin have reduced air gaps between the skin and cover and thus an increased cooling capacity. The AS pads provide the best fit due to direct contact with the hydrogel and skin surface. In comparison, the adjustable vest and head wrap of the KK maximized skin contact around the torso and head but was less effective over the lower body. The BL blankets lacked a tight fit for the upper blanket, but body weight maximized skin contact with the lower blanket.

Surface design and adhesion also impact heat transfer. The AS pad is rigid and therefore the skin side remains flat when liquid flows through it. Blanketrol covers are not rigid and liquid flow causes a quilting effect, and thus, some areas are in direct skin contact while others are not. Adhesion of the AS pads also increases heat transfer.

Table 2 indicates that each cover has advantages in different aspects of design. The AS gel pads have the advantages of higher thermal conductivity, tightness of fit, and adhesion to the skin. However, these advantages are offset by the increased surface areas of BL and KK and the higher cooling capacity of the Blanketrol III cooling pump unit. Thus, it is not surprising that there is little difference in heat loss, especially in the upper body, between the systems (Figs. 3 and 4). Given the similarity in heat loss between the systems, we cannot explain the greater decrease in core temperature (0.3–0.4°C) with AS, nor can we determine the clinical significance over a long cooling period of >24 hours currently recommended. Longer cooling periods in nonshivering subjects are required to address this question.

**Table 2.** Rankings of Cooling Pump and Cover Characteristics That Impact Heat Transfer

	AS	BL	KK
Cooling pump characteristics			
Outflow liquid temperature (°C)	4	4	4
Liquid flow rate (L/min)	1.7	2.3^*^	2.3^*^
Cover characteristics			
Surface area (% of BSA)	45%	75%	60%
Surface material (k)	↑	—	—
Tightness of fit	↑↑	—	↑
Adhesion	↑	—	—

↑↑, highest ranking; ↑, higher ranking; —, lowest ranking; ^*^Assumed based on the manufacturer's specifications; k, thermal conductivity.

AS, Arctic Sun; BL, blankets; KK, Kool Kit.

Practical factors should also be considered. The nonadhesive aspect of the BL and KK covers makes it easier to apply them to a patient and would allow easy access to any skin areas for monitoring or treatment of injuries. These covers can also be applied over open wounds where adhesive covers would be contraindicated. Also, the adhesive covers provided difficulty and discomfort during removal from the skin. Finally, it is also noteworthy that both the cooling pump unit and the covers are much less expensive for BL and KK compared with AS.

### Limitations

Some of the limitations of this study include subject demographics and the presence of shivering. The subjects were young healthy adults and may not be representative of the populations that often require TH. These patients are often older and have greater mass and surface area. Larger patients would be more difficult to cool compared with the subjects enrolled in this study. They may also have increased heat loss due to their increased body surface area. Future research should seek to confirm the results of this study by testing larger sample sizes and populations that are more representative of TH patients. Future studies should also aim to compare the performance of cooling systems when shivering is inhibited, as it will be more characteristic of clinical practice.

## Conclusion

Heat transfer capabilities of each system are dependent on the cooling pump unit and liquid-perfused covers. Both Arctic Sun and Blanketrol III cooling/pump units in this study had similar performances in their ability to reduce liquid temperature. However, the Blanketrol III unit has a greater flow rate and likely more cooling power. The AS pads have a limited surface area but have the advantage of high thermal conductive surface material, and tight fit due to the adhesive surface. The transient (∼10 minutes) advantage in heat removal with AS was not evident after about 20 minutes and thus represents a minimal advantage over extended periods of cooling (e.g., minimum of 24 hours) that are recommended in guidelines (Callaway et al., 2015a; Callaway et al., 2015b; Deakin et al., 2010).

The two BL blankets cover the largest surface area, but heat transfer was limited by its surface material, loose fit, and lack of adhesion. Finally, the size and tight fit of the KK gave it effective coverage around the torso and head, but the cover is also limited by its surface material, surface design, and lack of adhesion. However, the BL and KK covers were much easier to apply to the subjects. This provides the advantage over adhesive pads for the ability to check areas under the covers for injuries, treatment, and so on. Also, the AS adhesive covers provided difficulty and discomfort during removal. It is also noteworthy that both the cooling pump unit and the covers are less expensive for BL and KK compared with AS.
